# Pure Acute Subdural Hematoma of Cortical Non-mycotic Microaneurysmatic Etiology: A Report of Two Cases and a Literature Review

**DOI:** 10.7759/cureus.74387

**Published:** 2024-11-25

**Authors:** Énia Sousa, Ricardo Pestana, Ana Caleia, Catarina Barreira, Pedro Lima

**Affiliations:** 1 Neurological Surgery, Hospital Central do Funchal, Funchal, PRT

**Keywords:** acute subdural hematoma, brain hemorrhage, cortical microaneurysm, ruptured cerebral aneurysm, spontaneous brain hemorrhage

## Abstract

Pure acute subdural hematoma (ASDH) is an uncommon clinical presentation of ruptured intracranial aneurysms, and only rarely, the culprit is a cortical microaneurysm.Mortality can be high; thus, appropriate diagnosis and treatment are crucial. Due to its extreme rarity, there are no available guidelines.We aimed to describe two clinical cases of pure ASDH due to cortical microaneurysm rupture, and a literature review was performed.

A 33-year-old man, with no history of head trauma, was admitted with headache and left hemiparesis. Computed tomography (CT) showed right convexity ASDH, and CT angiography excluded intracranial vascular malformations. An emergent craniotomy was performed, and a microaneurysm was identified; the lesion was electrocoagulated and wrapped in muslin. The patient was discharged three weeks after with a modified Rankin Scale (mRS) of 1. A 58-year-old woman was admitted to the emergency room (ER) with a presumed history of head trauma. The Glasgow Coma Scale (GCS) was 4, and the left pupil was mydriatic. A CT scan showed a left convexity ASDH. Emergent decompressive craniotomy was performed, and the cortical surface beneath the hematoma revealed a microaneurysm that was clipped. The neurological status improved, but she perished 14 days after the procedure for nosocomial pneumoniae.

To date, few cases of pure ASDH due to cortical non-mycotic microaneurysm rupture were reported, including the two described in this article. Digital subtraction angiography (DSA) continues to be the gold standard for diagnosis. Nevertheless, the possibility of angiographically unvisualized lesions should be considered. The risk of rebleeding is high, and it foresees a worse outcome. Prompt diagnosis and treatment are imperative to achieve better outcomes, and the craniotomy must be extensive to expose a large area of the brain surface beneath the hematoma to assess a possible bleeding source. After treatment, the outcome is good in most published cases.

In patients presenting with nontraumatic ASDH, after the imageological exclusion of vascular intracranial malformations, the craniotomy for hematoma evacuation should be large to visualize all the brain surface beneath the hematoma to identify possible microaneurysm as the source of bleeding. To improve the outcome, the microaneurysm should be treated to prevent rebleeding.

## Introduction

Acute subdural hematomas (ASDH) are mostly caused by moderate to severe head trauma [1-5] due to the rupture of bridging veins in the subdural space [2]. Spontaneous ASDH is rare [1,2,4,6], accounting for only 2.6% of all cases [1]. The possible etiologies are extensive: the rupture of cortical arteries, aneurysm rupture, coagulopathies, vascular malformations, intracranial hypotension, neoplasms, and vascular inflammatory diseases, among others [1,4,5]. Spontaneous ASDH in association with subarachnoid hemorrhage (SAH) may be the clinical presentation of 0.5%-7.9% of cases of ruptured intracranial aneurysms [1-10]. However, pure ASDH, i.e., without concomitant SAH, is an extremely rare clinical presentation of ruptured intracranial aneurysms [1-4,7-9], with 45 cases published to date [3,7]; nevertheless, only rarely, the culprit aneurysm was a non-mycotic microaneurysm located in the cortex [7]. Mortality can be as high as 37.2%, so appropriate diagnosis and treatment are crucial [4]. Due to its extreme rarity, it is hard to elaborate on established guidelines; thus, treatment decision-making is based on personal experience [10]. In this report, we aimed to describe two clinical cases of pure ASDH due to non-mycotic cortical microaneurysm rupture and their clinical presentation, diagnosis workout, and surgical management. A literature review was also performed.

## Case presentation

Clinical case 1

**Figure 1:**
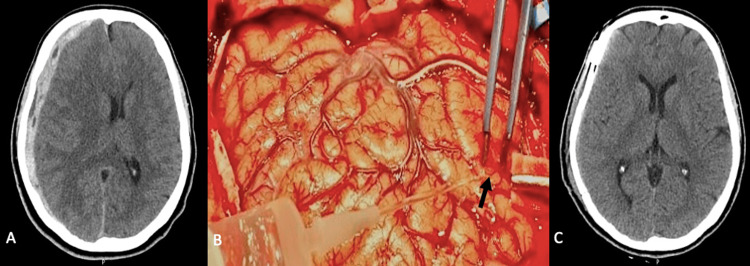
(A) Head CT scan with ASDH and midline shift. (B) Cortical microaneurysm (arrow). (C) Postoperative head CT scan. CT, computed tomography; ASDH, acute subdural hematoma

A 33-year-old man, with no relevant medical history, presented with sudden-onset right hemicrania headache, nausea, and vomiting after scuba diving. He had no history of head trauma. At observation, his Glasgow Coma Scale (GCS) was 15, and he presented with grade IV left hemiparesis. Head computed tomography (CT) showed right convexity ASDH with midline shift (Figure 1A), and CT angiography excluded intracranial vascular malformations. A large craniotomy was performed to evacuate the ASDH. After the inspection of the cortical surface, a microaneurysm was identified on the right posterior temporal cortical branch of the middle cerebral artery (MCA); there was bleeding after the suction of the covering clot (Figure 1B). The lesion was electrocoagulated and wrapped in muslin. The postoperative CT scan did not show any complications of the procedure (Figure 1C). He recovered from the previous deficits and was discharged three weeks after with a modified Rankin Scale (mRS) of 1.

Clinical case 2

**Figure 2:**
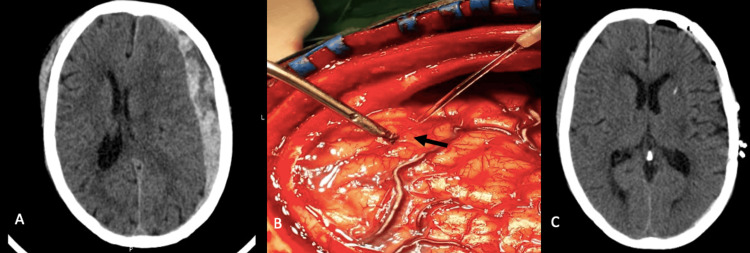
(A) Head CT scan with ASDH and midline shift. (B) Clipped cortical microaneurysm (arrow). (C) Postoperative head CT scan. CT, computed tomography; ASDH, acute subdural hematoma

A 58-year-old woman with a history of epilepsy and chronic alcoholism was admitted to the emergency room (ER) after being found unresponsive in her home, with a presumed history of head trauma. The GCS was 4, and she had anisocoria with mydriatic left pupil. A head CT scan showed a left convexity ASDH with a midline shift of >10 mm (Figure 2A). An emergent decompressive craniotomy was performed, and the ASDH was evacuated. The inspection of the cortical surface showed a microaneurysm of a cortical branch of MCA that was clipped (Figure 2B). Postoperative CT scan showed the complete drainage of the hematoma, without ischemic or hemorrhagic intracranial lesions, nor edema or midline shift (Figure 2C). The patient had progressive neurological improvement achieving a GCS of 11T at 12 days after the procedure. Due to the long-term invasive mechanical ventilation (IMV), she had complications of pneumoniae and ended up perishing 14 days after the procedure.

## Discussion

When evaluating nontraumatic ASDH, the differential diagnosis is extensive, and it is important to exclude underlying intracranial vascular malformations [1,2]. Most pure ASDH are presumed to be caused by a ruptured cortical artery, but the pathology and clinical features remain unclear [9], and even though this condition may be caused by cortical microaneurysm rupture, only 12 cases were reported to date [2,4,7,9,10], according to our literature review summarized in Table 1.

**Table 1:** Characteristics of patients with pure ASDH caused by non-mycotic cortical microaneurysm rupture. Adapted from Awaji et al. [4] and Verhey et al. [7]. MCA, middle cerebral artery; DSA, digital subtraction angiography; ND, not disclosed; ASDH, acute subdural hematoma

Reference	Sex	Age	Symptoms	Aneurysm location	Diagnosis	Treatment	Prognosis
Kurabe et al., 2010 [11]	Male	75	Headache and vomiting	Cortical branch of MCA	DSA	Hematoma evacuation and aneurysm clipping	ND
Boop et al., 1961 [12]	Male	37	Decreased level of consciousness and hemiparesis	Cortical branch of MCA	DSA	Hematoma evacuation and aneurysm resection	Good
Hori et al., 2005 [6]	Male	57	Headache, nausea, decreased level of consciousness, and oculomotor palsy	Precentral artery	Intraoperative	Hematoma evacuation and aneurysm clipping	Good
Sung et al., 2012 [2]	Male	58	Decreased level of consciousness	Cortical branch of MCA	DSA	Hematoma evacuation and aneurysm resection	Good
Gong et al., 2014 [8]	Male	43	Headache	Cortical branch of MCA	DSA	Hematoma evacuation and aneurysm resection	Good
Singla et al., 2014 [3]	Female	25	Decreased level of consciousness and hemiparesis	M5 branch of MCA	DSA	Hematoma evacuation and aneurysm clipping	Good
Awaji et al., 2016 [4]	Male	43	Headache and nausea	Cortical branch of MCA	Intraoperative	Hematoma evacuation and aneurysm clipping	Good
Verhey et al., 2018 [7]	Male	69	Headache	Cortical branch of MCA	DSA	Hematoma evacuation and aneurysm clipping	Good
Yanagawa et al., 2021 [9]	Male	63	Decreased level of consciousness	Anterior parietal artery	Intraoperative	Hematoma evacuation and aneurysm suturing	Good
	Female	85	Headache and decreased level of consciousness	Posterior parietal artery	Intraoperative	Hematoma evacuation and aneurysm suturing	Good
	Male	63	Decreased level of consciousness and hemiparesis	Angular artery	Intraoperative	Hematoma evacuation and aneurysm trapping	Good
Shekarchizadeh et al., 2017 [5]	Female	26	Headache and decreased level of consciousness	Distal anterior communicating artery	DSA	Aneurysm endovascular treatment	Good
Present cases, 2024	Male	33	Headache, nausea, vomiting, and hemiparesis	M4 branch of MCA	Intraoperative	Hematoma evacuation and aneurysm wrapping	Good
	Female	58	Decreased level of consciousness and oculomotor palsy	M4 branch of MCA	Intraoperative	Hematoma evacuation and aneurysm clipping	Bad

In these 14 published cases, including the two described in this article, of pure ASDH due to the rupture of non-mycotic cortical microaneurysm, there was a male predominance (10), contrary to the extensive published literature on ruptured aneurysms that has a clear female predominance. The median age was 57 (standard deviation: 17.7), keeping with the literature on ruptured aneurysms. The most common clinical presentation was a decreased level of consciousness (nine), followed by headache (eight), hemiparesis (four), nausea (three), vomiting (two), and oculomotor palsy (two). All these symptoms are related to the hematoma mass effect and consequent rise in intracranial pressure, thus not exclusive to a microaneurysmatic rupture.

Even with better imageology resources, the diagnosis of these lesions continues to be a dilemma in patients with trivial head trauma and with ASDH disproportionate to the energy of the trauma or in cases without a history of trauma but whose imaging studies do not demonstrate intracranial vascular lesions (occult cortical microaneurysm). Digital subtraction angiography (DSA) continues to be the gold standard for diagnosis; however, in patients with worsening neurological status, CT angiography may be an acceptable alternative because it saves time [3]. Even if a vascular abnormality is not identified in imaging examinations, the possibility of an angiographically unvisualized lesion should be considered [2]. In the first case presented, given the absence of a history of trauma, a CT angiography was performed, but the microaneurysm was only observed during surgery. In the second clinical case, given the presumed history of trauma, no other complementary diagnostic image examinations were requested. Nevertheless, in the literature review, all the patients who had DSA performed preoperatively were diagnosed with microaneurysm, reinforcing the importance of this diagnostic tool in such cases, given that the patient is neurologically stable to withhold a surgical evacuation of the hematoma.

Previous studies have found that the risk of rebleeding is high if proper hemostatic treatment is not performed for the cortical microaneurysm [9]. The treatment of the aneurysm eliminates the risk of rebleeding, a scenario that usually foresees the worse outcome; thus, prompt diagnosis and posterior treatment are imperative to achieve better outcomes [7]. In 13 cases, the treatment of the ASDH was surgical evacuation, with one being treated conservatively. In such cases, the craniotomy must be extended to expose a large area of the brain surface beneath the hematoma [6] to assess a possible bleeding source [2,9]. This is especially true in patients whose diagnostic examinations did not show an intracranial vascular malformation. The craniotomy in both our cases was large, a frontotemporoparietal craniotomy, allowing the evacuation of the hematoma and the evaluation of all the brain surface beneath the hemorrhage; this allowed for the intraoperative identification of the microaneurysms. Very often, these lesions can only be identified by microscope magnification, being recommended a careful evaluation of the brain cortex with this instrument [9].

All aneurysms were treated either by clipping (six), resection (three), suturing (two), trapping (one), electrocoagulation and muslin wrapping (one), or endovascular treatment (one). The outcome was good in 12, with one perishing due to other complications not related to the aneurysm treatment itself (the second clinical case in this report), and one report has not disclosed the outcome (Kurabe et al. [11]).

## Conclusions

In patients presenting with a nontraumatic ASDH, it is important to exclude intracranial vascular malformations, ideally by DSA, but in deteriorating patients, CT angiography can be an alternative. The craniotomy for hematoma evacuation should be large enough to visualize all the brain surface beneath the hematoma to identify possible microaneurysms as the source of bleeding, especially in those without intracranial vascular malformations in imaging examinations. The microscope can be an important tool to assess the brain surface and its vessels for microaneurysms. To improve the outcome, the microaneurysm should be treated as soon as possible to prevent rebleeding.
